# Government responses to gender-based violence during COVID-19

**DOI:** 10.3389/fgwh.2022.857345

**Published:** 2022-08-18

**Authors:** Rebecca Gordon, Nic Cheeseman, Sarah Rockowitz, Laura M. Stevens, Heather D. Flowe

**Affiliations:** ^1^School of Education and Social Sciences, University of the West of Scotland, Glasgow, United Kingdom; ^2^International Development Department, University of Birmingham, Edgbaston, United Kingdom; ^3^School of Psychology, University of Birmingham, Edgbaston, United Kingdom

**Keywords:** COVID-19, gender equality, gender-based violence, pandemic, policy, civil society

## Abstract

Gender-based violence (GBV) significantly and substantially threatens women's health. The COVID-19 pandemic has exacerbated existing risks and patterns of GBV. The impact of COVID-19 on GBV is not inevitable, however, and can be mediated by the policies of governments. In this study we developed the *Government GBV Response Index* to systematically examine how countries (*N* = 60) performed in response to the pandemic with respect to the government 1) enacting specific national-level GBV policy; 2) making dedicated COVID-19 specific funding available; and 3) adapting existing GBV responses to COVID-19 related restrictions and challenges. Most countries (*N* = 33) delivered fewer than two policy responses. We also performed rapid case study analyses to investigate what might contribute to countries having more comprehensive government policy. We find that civil society organizations played a key role in facilitating GBV policy during the pandemic, especially if they are well-funded and well-connected to the government, and if the country has a high-level government official responsible for gender issues.

## Introduction

Gender-based violence (GBV) is one of the greatest threats to women's health and wellbeing today. Although GBV is experienced by both men and women, women experience higher rates of repeat victimization and are much more likely to be seriously hurt or killed (1). Indeed, decades of research have demonstrated that violence against women is prevalent in all contexts; however, during emergencies there can be disruptions to protective structures and services that can lead to increasing levels of abuse and poor responses, exacerbating adverse health outcomes (2, 3). COVID-19 was particularly concerning in this regard due to social distancing, lockdowns and other restrictions of movement that put women and girls at greater risk of violence inside their own homes (4). Along with school closures, this meant that there were fewer opportunities to occupy safe spaces (5). Furthermore, the emotional and economic stress caused by the crisis is likely to have led to an increase in abuse (6, 7). These interrelated factors led UN Women (8) to describe GBV as a “shadow pandemic” occurring alongside COVID-19 worldwide, leading to estimates that there would be an additional 31 million cases of GBV globally by 6 months into the pandemic (9).

This may make it appear as if increases in GBV during the coronavirus pandemic would be inevitable. But, in reality, the impact of COVID-19 on GBV is not predetermined. To see why, it is important to start by acknowledging that COVID-19 itself has not *caused* GBV in any simplistic sense. Instead, lockdown measures implemented to slow transmission rates of COVID-19 during the pandemic have exacerbated existing risks and patterns of behavior (4). Moreover, it is likely that the impact of COVID-19 can and has been mediated by the policies of governments, the activities of non-governmental organizations (NGOs), civil society organizations (CSOs) and ordinary citizens. As is often the case, civil society plays a major role in affecting change in relation to GBV policies (10). One implication of this is that previous cuts to funding for relevant organizations prior to the pandemic likely influenced the nature and effectiveness of the response to GBV during COVID-19 (11).

Some governments reacted quickly and effectively to the threat of greater violence against women and girls, increasing funding for GBV services, safehouses, and hotlines, while stepping up messaging and communication on these issues (12, 13). However, in some cases governments diverted resources from GBV prevention and response, cut funding to organizations working in the sector and stifled free speech and dissent about these actions (14). In others, government policy was inconsistent and self-defeating, making statements and policies designed to respond positively to GBV while simultaneously reducing the funding for women's rights organizations (15). One common thread noted by researchers examining gender-based violence during the pandemic was that when government responses were swift and thorough, it offset the worst impacts of the pandemic, weakening the link between lockdowns and increasing GBV (16).

This is a critical point for two reasons. First, it serves as an important reminder not to be fatalistic or defeatist about the impact of health crises on GBV - they represent a massive challenge, but governments still have agency about how to respond. Relatedly, viewing the gendered pandemic in this way lays bare the culpability of governments, who have the opportunity to offset the greater risk women face during a pandemic but often, due to gender-insensitive systems and policies, actually serve to magnify the risk (17). Second, the positive impact of the measures implemented by gender-sensitive governments provides positive examples of “good basic practices” that policy makers and gender-activists can learn from - and can use to mobilize support for more progressive government responses in future. Rising GBV rates have not simply been organically triggered by pandemic lockdown conditions but have also, in many cases, occurred as a result of a lack of effective government response.

However, whilst this literature has acknowledged the importance of government responses to gender-based violence during health crises, thus far there have been limited comparative studies examining the way in which governments did respond to GBV during COVID-19. Therefore, our research sought to answer the question: What were government responses to GBV during the COVID-19 pandemic? Firstly, this article will explore the pre-existing research and literature on how governments responded to GBV during COVID-19, before presenting a detailed description of our methodological approach to exploring this phenomenon. We next present our findings and discussion of these findings before concluding with recommendations for policymakers and activists working in this space.

## An overview of existing research and understanding on government responses to GBV during COVID-19

Early on in the COVID-19 pandemic, there were calls for governments to make the prevention of violence against women and girls a key part of national emergency response plans, and for shelters and helplines to be included as essential services so they would remain funded and available for use (18). The World Health Organization made similar recommendations for government response plans to include essential services to address GBV, and also noted that health providers, health facilities, community members, and humanitarian response organizations must work to help mitigate GBV impacts arising from the pandemic (19). However, despite these calls, widespread closures of schools and support organizations, coupled with a decrease in government funding, has made protecting and responding to the needs of survivors difficult, and at times impossible, to achieve. For example, domestic violence organizations and safe shelters that have remained open have had an overwhelming amount of GBV cases in some instances, while in others the call numbers have decreased significantly because perpetrators are making it difficult for survivors to access protection and other essential services (20). Additionally, decreased funding owing to the economic fallout of the pandemic has caused many organizations to shut down their programmes and facilities (20).

One common policy that some countries enacted during lockdown was to ban the sale of alcohol altogether, as in India, the Philippines, and South Africa. Other countries, such as those in Europe and the Americas, left alcoholic beverages available, albeit with restricted sales hours in some locations (21). Due to lockdown restrictions, the limitations on alcohol sales were largely targeted at reducing incidents of domestic violence, as drinking in the home was often the only acceptable place of consumption. However, the ban on alcohol was also targeted at reducing other alcohol-related injuries, such as those from street violence, and to encourage compliance with social distancing measures (22). In South Africa, the alcohol ban was effective both in freeing up hospital beds for COVID patients and in making women feel safer, as their communities were less violent (23). Once the alcohol ban was lifted, emergency visits to hospitals doubled and domestic violence increased, taking up valuable healthcare resources in a country already struggling with providing adequate healthcare for all of its residents (23).

Research during this period also focused strongly on the role of civil society campaigns in putting pressure on the government to respond to rising rates of GBV. In Kenya, the coalition on violence against Women-Kenya urged the Health Cabinet Secretary to integrate GBV into the daily COVID-19 briefing, and a consortium of feminist organizations petitioned the government for a 30% allocation of COVID-19 funds to the GBV response (24). Ultimately, the pressure that GBV stakeholders and women's groups placed on the government to pay attention to rising GBV rates led to the late release of guidelines about health care for GBV survivors (25). UN agencies have also promoted global campaigns calling on governments to act on these issues with the message: “Fund, Prevent, Respond, Collect!” (18). In other cases, international NGOs have supported motivated political actors to act on their desires to address rising rates of GBV. For example, Oxfam in Malawi donated four motorcycles to women members of parliament to help them in rolling out campaigns to fight against GBV (26).

However, whilst there is growing evidence on how policy measures that were introduced impacted on GBV, and on the response of civil society mobilizing to campaign for improved governmental approaches to address these impacts, there is a lack of research that has systematically examined government responses on a global basis which our study seeks to address.

## Materials and methods

### Study design

In order to effectively critically evaluate government responses to GBV during the pandemic, we sought to utilize a comprehensive and globally used definition of the term: violence directed against a person because of their gender, which includes physical, sexual, psychological or economic harm or suffering (27). However, as noted above we acknowledge that most gender-based violence is inflicted on women and girls, by men.

#### Mapping government responses to GBV

To provide a reliable assessment of the global response to GBV since the start of the pandemic, and to highlight examples of good and bad practice, we conducted a rapid mapping of the actions of governments in 60 countries worldwide, with a stratified sample to ensure representation from different continents and from high-, middle- and low-income contexts. This sample of cases therefore reflects a broad cross-section of the global experience and provides insights into the extent to which county-level factors such as GDP and women's representation in parliament relate to the effectiveness of the government's response to the gendered impact of COVID-19 responses.

#### Rapid case studies

Based on our large-n analysis, we also conducted two rapid case studies to better understand the driving factors behind more comprehensive government responses to GBV during COVID-19. The purpose of these case studies were: to confirm our initial assessment of the government response and better understand the enablers and barriers to an effective government response.

### Sampling

#### Mapping government responses to GBV

Our decision to purposively sample 60 countries was based on a number of factors. Due to time and funding constraints, it was not possible to conduct the analysis for all countries impacted by COVID-19, and it is important to keep in mind - in addition to the caveats noted above - that our tracker represents a sample of around one-third of relevant cases. Nonetheless, we have been careful to stratify the sample to ensure representation from different continents, from high-, middle- and low-income contexts, and from states with very different population sizes and levels of social assistance.

**Figure 1:**
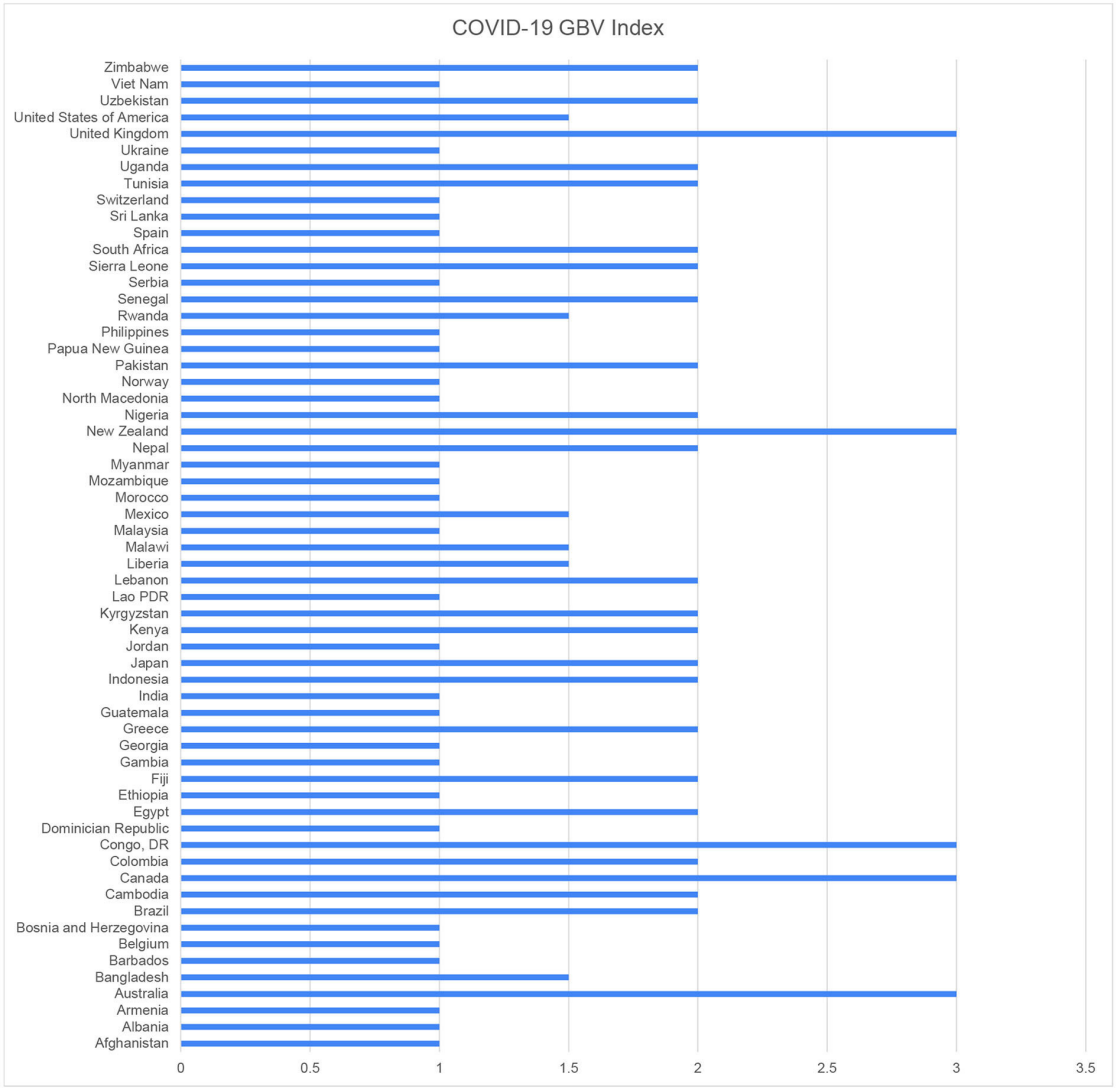
Government responses to GBV during COVID-19 overview.

It is important to note that we were careful to avoid falling into the trap of only collecting data on those countries in which it is easily available, and hence skewing the sample toward higher-income countries. Our sample therefore includes some of the poorest countries in the world, including the DRC, Malawi, Papua New Guinea, as well as countries with considerable conflict and instability such as Lebanon, Nigeria, and Myanmar. The full sample is shown in Figure 1 below. While more work needs to be done to confirm that our findings are repeated in the full set of cases, our sample was chosen to represent a reasonable approximation of the global context.

#### Rapid case studies

Our two rapid case studies were selected based on our analysis of the mapping of government responses. Both countries, the Democratic Republic of Congo (DRC) and New Zealand were chosen because both governments responded to all three measures on our mapping index (explained in more detail below) but have extremely different contextual factors that likely mediate government responses, including levels of GBV and government legislative and financial capacity. The logic of this approach is to show that even countries with limited resources can pro-actively respond to increases in GBV, and that many countries that could respond effectively fail to do so potentially due to a lack of political will, and to explore the impact of civil society actors and awareness raising. Therefore, these two case studies were considered to provide a useful insight into the factors which may support or hinder effective government responses to GBV during crises.

### Data collection

#### Mapping government responses to GBV

Data were collected for 60 countries in July 2021. However, data on government responses to the threat of rising GBV is not always well publicized or held in a central repository. It was therefore important to use a wide range of approaches to identify and check the robustness of data on gender policies and expenditure. Four complementary approaches were therefore utilized to collect the data that underpins the Government GBV Response Index. These included:

1. Consultation of existing data sets [e.g., (28)].
2. A set of Internet searches with various combinations of keywords, including COVID-19, coronavirus, GBV, domestic violence, policy, response, government funding, and all country names.

In cases where there was ambiguity or references but limited detail about potential government responses to GBV during COVID-19, the following steps were taken:

1. A search of news/press releases from each country's government website, where available.
2. A search for relevant national media coverage.

It is important to acknowledge that the index is calculated solely on the information and data available through these search terms which were conducted in English. In some cases, these were auto translated by Google, particularly when searches of news/press releases from government websites were being conducted. However, as a consequence of this is that there may be further information that was not found as part of this data collection approach. Additionally, as this was a rapid mapping exercise there may be information that was not easily accessible either through searches or through government websites, and so was not included. To reduce the risk of false negatives the data were shared with experts for verification and have proved to be robust. We are therefore confident of the reliability of the data that forms the backbone of this report.

Using these methods, we collected data on three responses that governments could make to try and reduce the impact on GBV. Although these policy responses are not exhaustive, they represent the main menu of options that governments utilized:

1. Were COVID-19 GBV-specific national protocols/policy/legislation introduced or was GBV mainstreamed within COVID-19 legislation or national responses?^1^
2. Was there COVID-specific GBV funding?
3. Was there a government supported COVID GBV service provision guidance response and adaptation/training/awareness campaign?

We created the **Government Response to GBV during COVID-19 Index** by awarding one point for each response (or in some cases 0.5 points – for example if some funds were allocated to GBV during COVID-19 but in relation to awareness raising rather than directly to support those responding to the crisis). On this basis, countries could score between 0 and 3, with 0 representing the weakest response to GBV during COVID-19 and 3 representing the most comprehensive, based on our indicators. It is important to note that the GBV Response Index effectively records official changes in policy and allocations of funding – it does not collect data on how effectively these strategies were implemented. We would therefore expect some variation between countries that have similar scores on the Index depending on how planned GBV responses were put into practice^2^. As we discuss in greater detail below, it is also important to keep in mind that given that there is no global dataset for the existing level of government activity across these three dimensions, it was not possible for us to take into account the different “starting position” that countries are likely to have when it comes to preventing GBV. Additionally, due to the challenges of data availability, we did not weight any of the components within our index, due to the likelihood of introducing distortions to the data; however, this would be a valuable avenue for future research.

#### Rapid case studies

The approach to these case studies was to conduct a rapid literature review of available policy and programming documentation, and to conduct five unstructured interviews across both countries with actors who had been involved in the response either within the government, or civil society/international organizations. These unstructured interviews took place over Zoom and were recorded and transcribed for analysis. Due to the ongoing pandemic, participants are not cited by name as they remain involved in the response.

## Results: Government responses to GBV during COVID-19

There is considerable variation among the 60 countries, demonstrating that comprehensive government responses to the gendered effect of COVID-19 – and other health emergencies – cannot be taken for granted (see Figure 1).

As evidenced by Table 1, on the positive side, no government scored 0 on the Index, meaning that every government in our sample put in place at least one of the measures set out above. Worryingly, however, only five countries were found to have enacted all three responses, and most countries were found to have enacted just one response. Overall, a majority of countries (33 as compared to 27) delivered fewer than two policy responses, meaning that a worryingly high number of governments failed to fully deliver two of three approaches that could have potentially limited the impact of the pandemic on GBV.

**Table 1:** Distribution of countries on the Government responses to GBV index.

**Government GBV response index**	**Number of countries**
0	0
1	27
1.5	6
2	21
2.5	1
3	5

Of course, which responses governments favored is as important a question as the number of strategies that governments put in place. As noted in Table 2, the most common response was to issue updated guidance and/or raise awareness to addressing higher risks of, and how to respond to rising GBV during COVID-19 (59 countries). This makes intuitive sense – updating guidelines and awareness raising campaigns can often be done by tweaking how existing funding and programming is delivered. This option therefore represents the path of least resistance – the strategy that is least likely to be opposed on the grounds of cost or time. The majority of the countries that appear to have deployed this strategy were low income and lower middle income contexts (including Bangladesh, Ethiopia, LAO PDR among others); however, there were a number of upper middle income countries that took this approach (including Albania, Armenia, and Jordan), although these tended to also be countries with Gender Inequality Index ranks below 50, which may indicate a more difficult legislative environment related to gender-specific responses. There were also some high-income contexts with high gender equality index ranking who appeared to take this strategy (e.g., Norway) that we explore below.

**Table 2:** Government policy responses to GBV during COVID-19.

**Policy response**	**Frequency of implementation**
Number of countries that introduced national specific GBV legislation/protocols which responded to COVID-19	23 (1 was mixed/limited and was coded as 0.5)
Number of countries that introduced funding to specifically address GBV during COVID-19	12 countries (3 had limited/mixed responses to funding)
Number of countries responded with updated guidance/increased or adapted service provision/awareness raising or communication campaigns	59 countries (3 had mixed responses which were coded as 0.5)

Twenty-three countries, some 38% of the sample, implemented pandemic-specific GBV legislation or national-level protocols. In some ways this is a surprisingly positive finding. Taking the time to design and pass new legislation and/or national-level protocols amidst a pandemic demonstrates that these governments were both aware of the gendered impact of the pandemic and willing to take steps to prioritize a policy response. For example, in Pakistan, the national government integrated measures to address violence against women and girls under the Socioeconomic Impact Plan and Pakistan's Preparedness Response Plan for COVID-19. Additionally, Provincial Governments have incorporated gender responsive planning guidelines with a specific focus on socioeconomic impacts of COVID-19 (28). In Lebanon, the National Commission for Lebanese Women issued a press release on amendments to the Domestic Violence Bill to promote protections for women and children that address increasing domestic violence as a result of the country's economic crisis and lockdown measures due to COVID-19 (28). In Brazil, new legislation (Law N.14022) was introduced to extend protective measures for women during the pandemic (28).

However, it is important to also recognize the shortcomings of government responses during this period. Most notably, we only found evidence of 12 countries that introduced new funding to finance GBV prevention activities. Countries that deployed this strategy included Australia, Bosnia and Herzegovina, Canada, DRC, Kenya, New Zealand, UK, USA, Uzbekistan and Zimbabwe. These are mostly (although importantly not all) high-income contexts, which suggests that in a number of cases the introduction of new measures was likely hampered by a lack of funds. In the case of the DRC and Zimbabwe this was clearly not the case, and we explore GBV responses in the context of low-income countries in greater depth below.

The full evaluation for each response for each country, and a justification for the score given, can be found in the data files for this project. When interpreting these scores, it is important to keep in mind that in some countries that had already established extremely comprehensive GBV laws prior to the pandemic there was no attempt to introduce new COVID-specific legislation as existing legal frameworks were seen to be sufficient. In some of these cases, such as Norway, we consider that this reflected a genuine sense that existing legislation was sufficient, rather than a derogation of duty – especially as the Norwegian government were proactive in providing donor support to low-income countries to address GBV during the pandemic (29). As a result, there are some cases in which governments that did not develop new legislation or funding did seek to meaningfully adapt and utilize existing laws and funding mechanisms to effectively address GBV during the pandemic. This was not the case, everywhere, however, which raises the question of what explains variation in government responses to GBV increases?

## Discussion: What explains governmental GBV responses?

There are many factors that might influence governmental responses to GBV at any point, and particularly during crises. Once we developed the Index, we explored whether there were any patterns that could explain the main factors that make it more likely governments respond proactively. Interviews as part of our case studies discussed below suggested that one important factor was the presence of active civil society organizations able to campaign on gender issues because they had direct contact and lines of communication to the relevant government agencies (either pre-existing or established at the outset of the pandemic). This meant they could raise awareness and put pressure on political leaders to take stronger action on GBV. For example, in March 2020, the Agency for Gender Equality Bosnia and Herzegovina (BiH) conducted a survey with Civil Society Organizations (CSOs) running shelters in BiH. Based on CSO feedback the Agency developed the Plan of Intervention measures to support CSOs running shelters, which became an integral part of the Plan of interventions of the Ministry of Human Rights and Refugees in BiH (28).

We explore this further in the final part of the paper, but in order to make sure that this “civil society awareness raising effect” is not simply a by-product of countries in which the conditions are more suitable – and to better understand the different forces that shape gender-sensitive policies – we first look at three other factors that the literature and past experience suggest may be related to government policy toward GBV: government income, on the basis that leaders with more resources have more room for maneuver, existing levels of gender inequality, on the basis that these are likely to reflect the position of women in society and the capacity of anti-GBV groups to effectively mobilize, and the representation of women within the political system, on the basis that countries with more women in prominent positions may be able to galvanize greater support for a gender-sensitive response from within the political elite (30). Levels of violence pre-pandemic may also be related to government policy toward GBV. However, due to the lack of systematic data collection regarding GBV worldwide and differences in reporting rates between countries, pre-pandemic violence could not be correlated against this Index.

**Figure 2:**
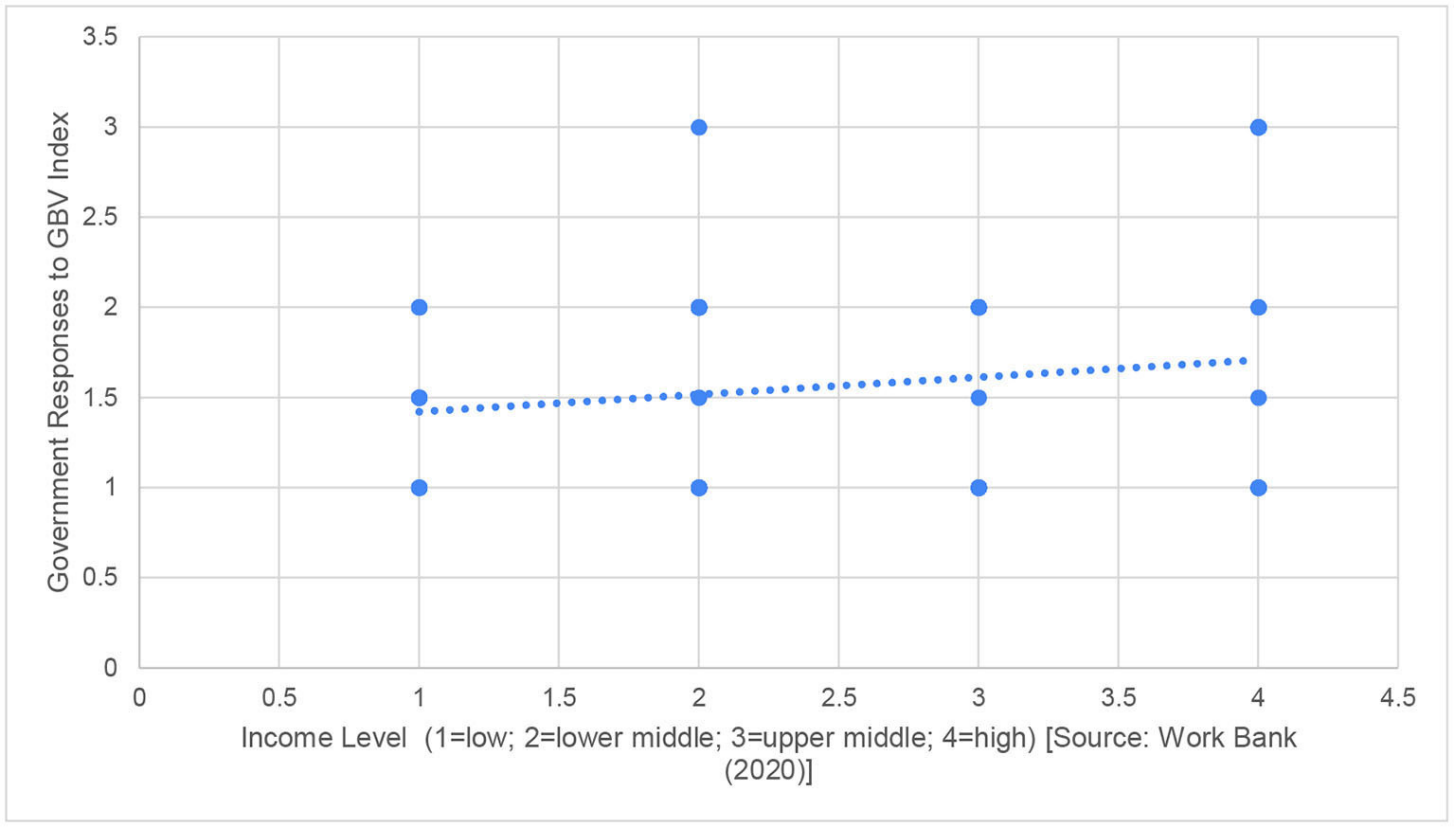
Comparing income level to government responses to GBV during COVID-19.

In each case, we illustrate the presence (or absence) of the relationship by plotting the Government Responses to GBV Index against the relevant factor, as in Figure 2^3^. In each case, a rising diagonal line from bottom left to top right indicates the presence of a strong relationship. Where Figure 2 is concerned, we look at the income level of the government/country concerned, using the World Bank classification that divides countries up into low, lower middle, upper middle-, and high-income countries (31). The graph illustrates that there is a slight increase in how countries perform on the Government Responses to GBV Index as they become wealthier, but this relationship is not statistically significant (Spearman's rho = −0.078, *p* = 0.551). In other words, there is no evidence from these data that wealth was the key determining factor that shaped gender-sensitive responses to the pandemic.

*Income Level (1* = *low, 2* = *lower middle, 3* = *upper middle, 4* = *high) [Source:* (32)*]*^4^

**Figure 3:**
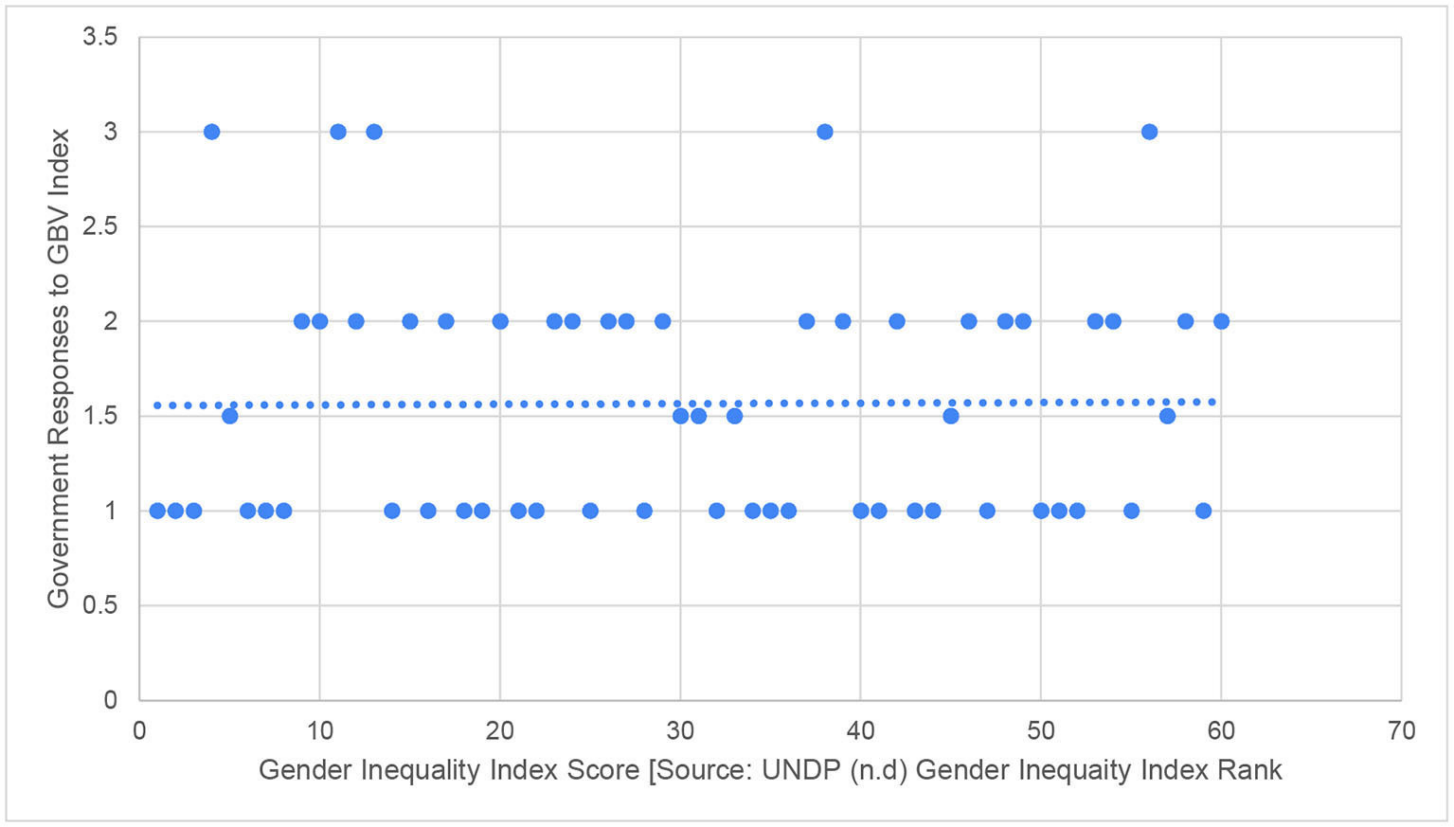
Comparing gender inequality index to government responses to GBV during COVID-19.

When we move to look at pre-existing levels of gender inequality, the relationship is even weaker. As depicted in Figure 3, there was no statistically significant relationship between gender inequality and Government Response to GBV during the pandemic (Spearman's rho = 0.110, *p* =0.41). Many countries with higher levels of gender inequality performed as well, or the same, as countries with lower levels. Gender inequality is here measured using the UNDP Gender Inequality Index, which takes into account inequality in reproductive health, empowerment and economic status (33). We have already discussed the point that some countries, such as Norway, may have implemented fewer measures in response to COVID-19 as they already had strong provision, which may help to explain the absence of a relationship in some cases, but even if we exclude these exceptional performers the relationship is marginal.

*Gender Inequality Index Score [Source: UNDP* (33) *Gender Inequality Index Rank]*.

**Figure 4:**
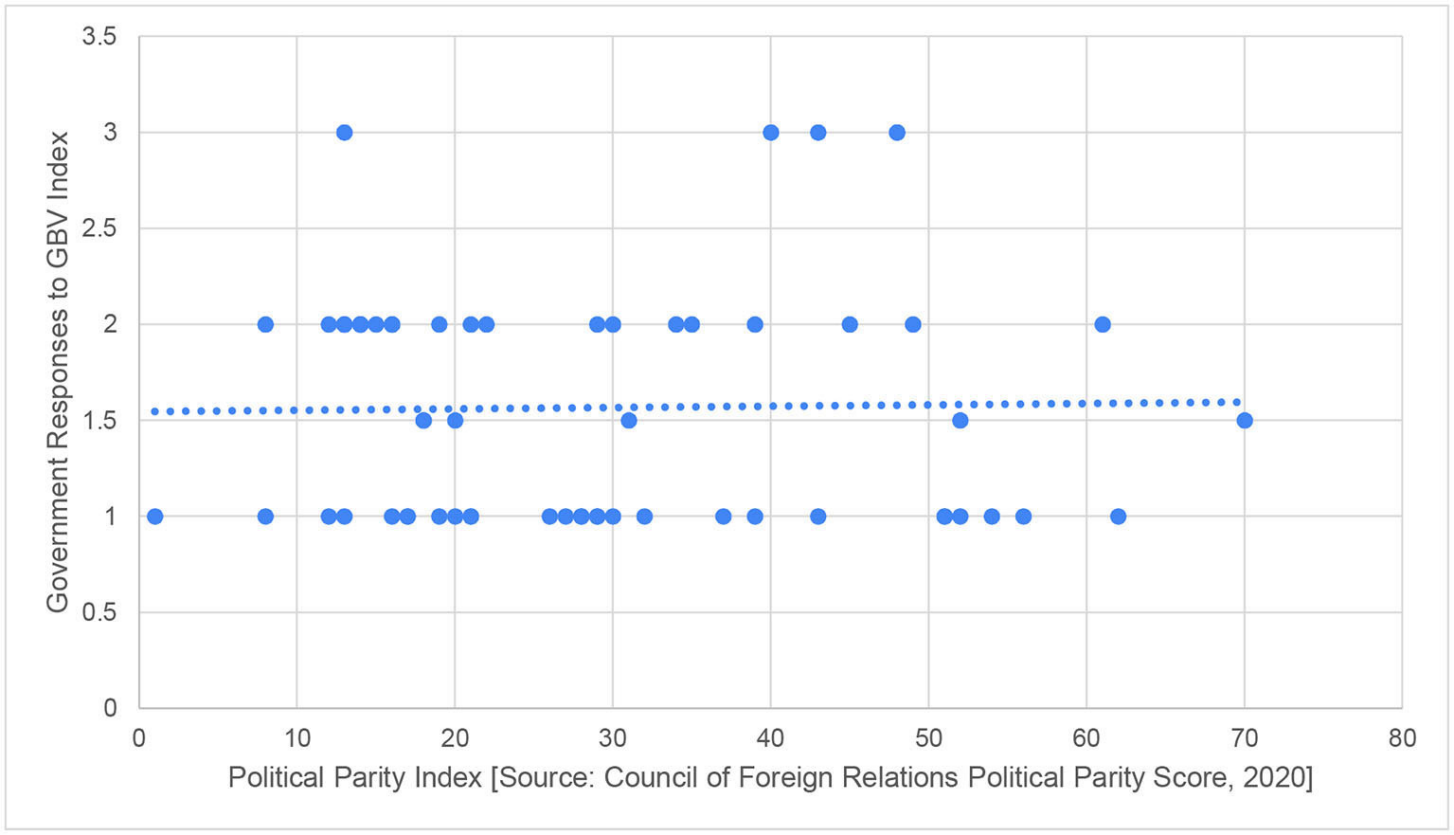
Comparing political parity index to government responses to GBV during COVID-19.

Thirdly, we consider whether the representation of women within political roles can lead to more comprehensive and effective policies related to gender issues, including GBV. To do this, we explored whether there is any link between a country's political parity score and their GBV response during COVID-19. The political parity score is an aggregate measure of the representation of women in a country's government, and hence, measures the likelihood that women leaders can shape policy. Again, the relationship was not statistically significant (Spearman's rho = −0.07, *p* = 0.583). As can be seen in Figure 4, as countries with low levels of political parity have had similar responses to the pandemic as those with high levels.

*Political Parity Index [Source: Council of Foreign Relations Political Parity Score*, (34)*]*.

Given that three of the most likely factors we identified are not statistically significantly related to how governments responded to the threat of rising GBV during the pandemic, it is clear that the explanation lies elsewhere. While this study cannot go into depth on every possible driver of government policy, there is considerable evidence that the role of civil society and non-governmental organizations, and their ability to galvanize political governments into action (10), has played an important role in ensuring that governments put gender-sensitive responses to the pandemic on the agenda. Although pre-existing data which maps the robustness of civil society was not consistently available to be able to explore this in relation to our Index, research has demonstrated that civil society has been considered to improve outlines in relation to gender-based violence directly, as a result of service provision (35), as well as playing an important role for monitoring and pressuring governments into action (36).

### Analysis of our rapid case studies: The importance of civil society actors and campaigns

A trend that quickly became apparent in the mapping of government responses, as mentioned above, was the importance of active and well-connected civil society organizations. As well as evidence of this emerging for the cases of Bosnia and Herzegovina and Kenya, there were multiple other examples in which civil society organizations – most notably women's movements and organizations – and international NGOs and UN agencies played a key role in galvanizing and supporting government responses. For example, the Ministry of Women's Affairs in Cambodia collaborated closely with NGOs and donor agencies to develop and distribute communication materials focused on GBV and COVID-19, collect data and document the impact of COVID-19 on GBV. It has also collaborated with UNFPA to develop appropriate response measures based on their study on the impacts of COVID-19 in the country (28).

In the Philippines, a particularly difficult context since the election of the populist President Rodrigo Duterte, civil society actors including women's rights organizations worked with progressive legislators to push for a bill that would criminalize child marriage. The legislation passed the Senate in November 2020 and received the support of the House of Representatives in September 2021 – becoming law – protecting young girls from a worrying increase in child marriages during the pandemic (37). Similarly, the Egyptian government was also supported by UN Women, UNFPA and other actors, to update its Standard Operating procedures in order to better address the pandemic (ibid). Our rapid case studies (Boxes 1, 2) explore these factors in more detail.

Box 1 **Aotearoa New Zealand.**

Aotearoa New Zealand has one of the highest rates of domestic violence in high-income contexts, with one in three women reporting physical abuse and one in two reporting psychological abuse from an intimate partner in their lifetime (38). However, New Zealand also had comprehensive and progressive pre-existing legislation to tackle GBV, in particular domestic violence. For example, The Domestic Violence - Victims Protection Act (2018) was a global landmark for holding workplaces accountable for safeguarding survivors of violence (39).

The response by the Government in New Zealand to GBV during COVID-19 was comprehensive with the introduction of new national protocols (40). Guidelines for the Family Violence and Sexual Violence workforce for COVID-19 were introduced and continually updated, so that family and sexual violence crisis workers could continue operating safely. The government also provided a comprehensive information site related to COVID-19 and GBV, with updated helplines and organizations for citizens to engage with to support with family and sexual violence prevention (41).

Targeted funding was also introduced; on 31st May 2020, the Minister for Women announced a 1 million NZD fund for organizations that support women and girls as part of the Government's COVID-19 response. It operated in the form of a one-off grant to support these organizations in the short term, but the funding was later doubled to 2 million NZD due to the level of demand. Finally, this was incorporated into mainstream funding structures, with an additional 200 million NZD allocated in the budget to respond to increased rates of violence during COVID-19 (28).

It is important to understand the facilitating and enabling factors which supported this comparatively comprehensive response. One factor mentioned was the fact that there was a **collaborative set-up already in place**. The Joint Business Venture Unit (JBVU) was set up by the undersecretary to co-ordinate ten ministries to have a unified response to family and sexual violence. The creation of one access point to multiple ministries (including health, education and others) meant that NGOs in the sector had a well-established line of communication to respond to the changing needs. During the first lockdown, open zoom meetings instigated by NGOs within the sector were joined by the JBVU to share information on what was happening on the ground to best coordinate the government-level response. The JBVU also offered practical action on policies and responses. Those within the sector agreed that public servants within the JVBU were open and responsive to their suggestions and willing to collaborate, acknowledging the unique situation and recognizing the limits of their expertise in this situation. The JVBU were already working across ministries to get more funding for the sector overall, and this allowed them to pivot during COVID-19 to provide one off grants to organizations. These were flexible and many of the usual deliverables and reporting were scrapped in order to enable organizations to effectively respond to the crisis. This amplified the high trust environment between the government and organizations working within the sector.

However, whilst the JVBU and the sector had a strong working relationship during the pandemic, there were challenges within the wider government, namely barriers to getting onto pandemic committees, which meant that the GBV/SRV lens was missing from the centralized crisis response. Some felt that this could have been addressed if there had been a specific cluster within the emergency response focused on GBV as often seen in other crises.

**NGOs were also pro-active and coordinated in their approach to raising issues with the government**. Pre-pandemic, the family and sexual violence sector had begun to work more closely together prior to this period, holding a joint conference in November 2019. This meant that there were good pre-existing relationships and a collaborative mindset which were activated quickly during COVID-19. This coordinated collaboration led to the online meetings between those in the sector and the government mentioned above. NGOs also set up sub-committees (which are still in operation) to focus on different issues and consistently share information with the government.

Finally, **pre-existing experience of adapting and responding to the Christchurch earthquake** was considered to have laid a foundation for approaches to crisis response. Christchurch had highlighted that responses to GBV during emergencies were not integrated within the wider response; although this was not ‘solved' by COVID, there was strategic learning from this response. For example, the government had recognized that communities know what to do best and how to respond, and so the learning from this was shared in multiple ways. In addition, the importance of pre-disaster relationships between the police, civil defense and women's refuges was highlighted during this event (42). Those in government also reflected on the fact that this had already stress-tested some of the issues that arose during COVID-19.

However, it was also widely acknowledged that whilst the response was driven and facilitated by an active civil society sector, and openness and willingness to listen from government, that a continued lack of funding and capacity of the family and sexual violence sector was a remaining barrier to a comprehensive response to GBV during the crisis, and beyond.

Box 2 **Democratic Republic of Congo.**

The DRC is known to have a high prevalence of GBV; 68% of women report lifetime exposure to physical, sexual or emotional violence (17). The Ebola epidemic (2018-2020) saw an increase in the reported risk and experience of GBV, both due to the fact that gender norms became further entrenched and because a fear of catching Ebola meant that women and girls experiencing abuse did not access services. The challenges were exacerbated by a lack of financial resources available to women and girls, and a lack of communication from the government (ibid). There were also reports of increased sexual and gender-based violence nationally during COVID-19, which were particularly severe in North Kivu and Goma, with school closures and financial difficulties being argued to put girls at increased risk of abuse (43).

Violence against women and girls was specifically included in the national programme to address COVID-19, *Programme Multisectoriel D'Urgence D'aténuation des impacts de la Covid-19 en RDC* with specific outputs, indicators, activities and a dedicated budget (28). This plan was introduced to address the effects of the pandemic, in particular economic stability and recovery but also for civil society working to address other areas, including GBV. Additionally, an online network of psychologists and social workers was set up to provide psychosocial support to GBV and COVID-19 survivors and to refer them to appropriate services, which was accompanied by increased awareness campaigns at the national, regional and community level (28).

The **well-coordinated responses from organizations working in this sector** had an important role to play in highlighting the adjustments and additional services that were needed. For example, UNFPA worked to provide a ‘one stop center' to help survivors of violence and worked with the government to put in place a helpline for those who could not physically access services. Similar to New Zealand, there was a pre-existing set-up in place coordinating stakeholders working on GBV, the GBV sub-cluster. During COVID-19, this was adapted to meet online and worked as a way to coordinate responses to GBV during the pandemic.

Those we spoke to who worked on these issues felt that **the government were open minded and receptive** to addressing the challenges related to GBV during COVID-19, and finding solutions to these issues. There were a number of key stakeholders within the government who played an important role in ensuring this comparatively comprehensive response, including those in the Ministry of Gender, Family and Children, the special adviser to the president on sexual violence of youth, the Minister of Health and the First Lady. There were a number of formal and informal conversations, with roundtables to discuss the response and getting the agenda set.

However, it was also noted that the government priority was responding to COVID, and whilst there were increases in funding to GBV services on paper, it is not clear whether these have been realized in practice. Indeed, researchers have emphasized that broader contextual issues, particularly related to funding, are likely to mediate government responses to GBV during COVID-19 (13).

## Conclusion

The results of the policy analysis indicate that civil society and non-governmental organizations had an important role in ensuring governments enacted gender-sensitive responses during the pandemic. Organizations that have strong links and communications with the government were particularly well placed to promote and enable political governments to take action. In particular, the results suggest that countries with previous experience of responding to emergencies (e.g., the Christchurch earthquake, the Ebola crisis) were especially well placed and could take effective action because there was capacity and precedence in how to respond and awareness of what would be needed to prevent and respond to GBV during the pandemic. Further, there was evidence that countries that have a dedicated minister (or person within a ministry) for GBV were particularly well-placed to respond. Having said this, there were active civil society actors and campaigns in all of the countries in the dataset, and there is a large amount of evidence (including that noted above) of their important role in adapting and responding to GBV during the crisis. Our results suggest that while civil society organizations are important to ensuring more comprehensive government policy responses, these organizations must be well funded and supported in order to make inroads and put firm pressure on political actors.

Our data provide detailed information about government policies enacted to control levels of GBV during the pandemic, which enabled us to compare countries and analyse policy responses to GBV during COVID-19. However, the data included only countries in which data were available regarding government GBV policy during COVID-19. As such, the data may not be representative of countries in which GBV government policy enactments are unknown. Therefore, inferences about GBV policy are limited to those countries in which information was available and accessible. Finally, information about the impact of government policy on GBV levels dur ing the pandemic remain unknown because GBV tends to be underreported, especially during emergencies such as the COVID-19 pandemic. There is therefore a need for governments to make greater investments in national statistical capacity to systematically collect and analyse gender data and use it to monitor and inform policy interventions.

## Data availability statement

The raw data supporting the conclusions of this article will be made available by the authors, without undue reservation.

## Ethics statement

The studies involving human participants were reviewed and approved by University of Birmingham. Written informed consent for participation was not required for this study in accordance with the national legislation and the institutional requirements.

## Author contributions

RG co-designed the research, undertook the data collection, and led the writing and analysis. NC co-designed the research and supported the writing and analysis. SR and LS undertook a literature review to support the article and supported the writing and analysis. HF supported the writing and analysis. All authors contributed to the article and approved the submitted version.

## Funding

This research was funded by a grant award from the Institute for Global Innovation at the University of Birmingham.

## Conflict of interest

The authors declare that the research was conducted in the absence of any commercial or financial relationships that could be construed as a potential conflict of interest.

## Publisher's note

All claims expressed in this article are solely those of the authors and do not necessarily represent those of their affiliated organizations, or those of the publisher, the editors and the reviewers. Any product that may be evaluated in this article, or claim that may be made by its manufacturer, is not guaranteed or endorsed by the publisher.

## Footnotes

^1^This related to Protocol/Policy/Legislation that specifically sought to address/mitigate rising rates of violence, rather than solely protocols to ensure the continued functioning of existing services, which were covered under the third response. When GBV was mainstreamed within COVID-19 legislation, this was considered to be a specific GBV-related government response to COVID-19 and so this was coded as 1.

^2^It is also worth acknowledging that in some cases government communication of their responses was more widely available than others. Therefore, there may have been government responses that were not clearly or widely communicated that this database does not capture.

^3^As noted above, it is not possible to take into account initial levels of support for GBV related interventions because there is not a dataset that collects this information for our set of cases. To the best of our knowledge, the closest dataset that exists is Htun and Weldon (10), but this only goes up to 2005, does not include all of our cases, and focuses on CEDAW ratification and regional level agreements, rather than levels of domestic funding and legislation.

^4^Our decision to use the World Bank classifications was due to the consideration of their greater stability during a period of economic fluctuation for many countries, which may have influenced the accuracy of this analysis were we to use other indicators, such as GDP per capita.
